# Bacteriological Spectrum and Antibiotic Utilization in Community-Acquired Pneumonia: An Observational Study at a Tertiary Care Center in Western Uttar Pradesh, India

**DOI:** 10.7759/cureus.113754

**Published:** 2026-07-31

**Authors:** Mamta Yadav, Mani Bharti, Ashwini Nigam, Yogesh Goyal

**Affiliations:** 1 Pharmacology and Therapeutics, Sarojini Naidu Medical College, Agra, IND; 2 Pharmacology and Therapeutics, Government Institute of Medical Sciences, Greater Noida, IND; 3 Medicine, Sarojini Naidu Medical College, Agra, IND; 4 Pharmacology and Therapeutics, Autonomous State Medical College, Firozabad, IND

**Keywords:** antibiotic resistance, antibiotic susceptibility, antimicrobial susceptibility, antimicrobial utilization, bacterial profile, bacteriological profile, community-acquired pneumonia, defined daily dose, sputum culture

## Abstract

Background and aim: Community-acquired pneumonia (CAP) is a leading cause of hospitalization and infectious disease mortality. Regional data on pathogen distribution, antimicrobial susceptibility, and antibiotic utilization patterns are essential for rational prescribing. The COVID-19 pandemic has further altered the epidemiology of respiratory infections and antibiotic prescribing practices in many settings. This study aimed to characterize the bacteriological profile, antibiotic susceptibility patterns, and antimicrobial utilization trends, including defined daily dose (DDD) and antibiotic consumption index (ACI), in hospitalized CAP patients at a tertiary care center.

Methods: A prospective observational study was conducted from September 2019 to March 2020 at Sarojini Naidu Medical College, Agra, involving 130 adult CAP patients. Sputum cultures and Kirby-Bauer disk diffusion susceptibility testing were performed. Antibiotic prescriptions were analyzed using the Anatomical Therapeutic Chemical (ATC)/DDD methodology, and ACI was calculated per 100 bed-days.

Results: A causative organism was identified in 101 of 130 cases (77.6%). *Streptococcus pneumoniae *(n=40, 30.8%) was the most common pathogen, followed by *Klebsiella pneumoniae *(n=27, 20.8%), *Escherichia coli *(n=24, 18.5%), *Staphylococcus aureus* (n=8, 6.2%), and *Pseudomonas aeruginosa *(n=2, 1.5%). Linezolid (100%) and carbapenems demonstrated the highest sensitivity; ampicillin and amoxicillin-clavulanic acid showed the highest resistance. Amoxicillin-clavulanic acid was the predominant empirical antibiotic (n=73, 56.2%; ACI: 14.4 DDD/100 bed-days), while levofloxacin was the most prescribed definitive agent (n=33, 25.4%; ACI: 14.8 DDD/100 bed-days). A paired analysis of antibiotics with complete empirical and definitive tracking data (n=7) showed no statistically significant overall shift in consumption volume (paired t-test, p=0.5948), though prescribing shifted markedly from frontline oral agents toward restricted broad-spectrum agents. Poly-antibiotic prescriptions were used in 115 of 130 patients (88.4%).

Conclusions: A significant proportion of isolates demonstrated resistance to commonly used antibiotics such as beta-lactams. *Streptococcus pneumoniae* remains the dominant pathogen, but the substantial burden of Gram-negative organisms with high resistance to first-line agents underscores the need for culture-guided therapy.

## Introduction

Community-acquired pneumonia (CAP) is among the leading infectious causes of hospitalization and mortality worldwide, affecting patients across all age groups but particularly those who are elderly or have underlying comorbidities. Similar to the study by File Jr and Ramirez [1], Anderson and Feldman also demonstrated the substantial global burden of CAP, including cardiovascular complications of invasive pneumococcal disease [2]. Bozkurt et al. estimated that CAP accounts for 20-30% of infectious disease-related admissions at tertiary care hospitals, imposing a significant burden on healthcare systems in both developed and developing nations [3]. Despite advances in antimicrobial therapy, Torres et al. highlighted that CAP remains a significant clinical challenge owing to the emergence of antimicrobial resistance (AMR) and heterogeneity in pathogen distribution across geographic regions [4].

The etiology of CAP varies considerably between settings. While *Streptococcus pneumoniae* remains the most commonly implicated bacterium globally, Gram-negative organisms such as *Klebsiella pneumoniae* and *Escherichia coli* are increasingly identified in tertiary care settings, particularly in South Asia. Shah et al. reported a high proportion of Gram-negative isolates in hospitalized Indian CAP patients, a finding corroborated by Goel et al. from an intensive care setting in North India [5,6]. This evolving microbiological landscape makes empirical prescribing complex and underscores the importance of local surveillance data, as emphasized by Gupta et al. in the Joint Indian Chest Society (ICS)/National College of Chest Physicians (India) NCCP(I) guidelines [7].

Simultaneously, irrational antibiotic prescribing - characterized by overuse of broad-spectrum agents, prolonged therapy durations, and high rates of polypharmacy - has accelerated AMR, increased healthcare costs, and prolonged hospitalization. Ginsburg and Klugman argued that the COVID-19 pandemic has further compounded this problem by driving widespread antibiotic use without adequate microbiological justification [8]. Drug utilization studies using standardized tools such as the WHO Anatomical Therapeutic Chemical/defined daily dose (ATC/DDD) classification and the antibiotic consumption index (ACI) allow objective, internationally comparable quantification of antibiotic use, as reviewed by Meena and Jayanthi [9].

Recent global events, including the COVID-19 pandemic, have further altered infection epidemiology, host immunity, and prescribing practices, adding complexity to CAP management. Griffin et al. documented sustained shifts in pneumonia hospitalization patterns even following a decade of pneumococcal vaccination, reflecting the dynamic nature of CAP epidemiology [10]. Torres et al. further highlighted that secondary bacterial infections during the COVID-19 era have significantly modified the pathogen profile [11]. Region-specific data integrating both the microbiological profile and drug utilization patterns remain scarce in many parts of India, including Western Uttar Pradesh.

Several studies have evaluated the microbiological profile or antimicrobial susceptibility patterns of CAP; few have simultaneously assessed antibiotic utilization using standardized WHO indicators such as defined daily dose (DDD) and antibiotic consumption index (ACI). Moreover, data from tertiary care centers in North India integrating bacteriological profile, antimicrobial susceptibility, and quantitative antibiotic consumption are limited.

## Materials and methods

Study design and setting

A prospective observational study was conducted from September 2019 to March 2020 at the Department of Medicine and Pharmacology, Sarojini Naidu Medical College, Agra, Uttar Pradesh. The study received approval from the Institutional Ethics Committee and Scientific Review Board and was registered with the Clinical Trials Registry of India (#CTRI/2018/02/011788). Written informed consent was obtained from all participants.

Study population

The study population included adult patients (≥18 years) of both sexes, admitted to the medicine ward of the hospital. Patients diagnosed with CAP based on clinical and radiological criteria were enrolled. CAP was defined as the presence of new radiographic pulmonary infiltrate in a patient who was not hospitalized or residing in a healthcare facility for ≥14 days before symptom onset.

Exclusion Criteria

Patients already receiving antibiotic therapy for >7 days prior to enrollment, hospital-acquired or ventilator-associated pneumonia, immunocompromised states (HIV, organ transplant, systemic steroids), active tuberculosis, lung malignancy, or patients who died or were lost to follow-up before discharge were excluded from this study. Of 166 initially enrolled patients, 24 discontinued treatment prematurely and 12 died during treatment. Prescription records from the remaining 130 patients were included in the final analysis.

Microbiological analysis

Sputum samples were collected prior to antibiotic initiation. Gram staining was performed on all samples to assess specimen quality (≥25 polymorphonuclear leukocytes and <10 epithelial cells per low-power field). Adequate samples were cultured on blood agar, chocolate agar, and MacConkey agar under standard conditions. Bacterial pathogens were identified by colony morphology, Gram stain characteristics, and standard biochemical tests.

Antibiotic susceptibility testing

Antimicrobial susceptibility testing was performed using the Kirby-Bauer disk diffusion method following Clinical and Laboratory Standards Institute (CLSI) guidelines [12]. Results were categorized as sensitive (S), intermediate (I), or resistant (R) for ampicillin, amoxicillin-clavulanic acid, piperacillin-tazobactam, ceftriaxone, ciprofloxacin, levofloxacin, doxycycline, azithromycin, amikacin, gentamicin, imipenem, meropenem, and linezolid.

Drug utilization analysis

Antimicrobial prescriptions were recorded at three time-points as follows: admission (empirical therapy), day three post-culture-sensitivity report (definitive therapy), and at discharge. Drug consumption was quantified using the WHO ATC/DDD methodology as reviewed by Meena and Jayanthi [9]. The number of DDDs was calculated as follows: number of DDDs = total g used ÷ DDD value (g). The ACI (DDD/100 bed-days) was calculated as follows: ACI = (number of DDDs/total bed-days)×100 by the WHO Collaborating Center for Drug Statistics Methodology [13]. Prescribing indicators including mean drugs per prescription, percentage prescribed by generic name, and percentage of intravenous prescriptions were also assessed.

Statistical analysis

Data were entered in Microsoft Excel (Redmond, WA: Microsoft Corp.) and analyzed using SPSS version 27.0 (Armonk, NY: IBM Corp.). Categorical variables were expressed as frequencies and percentages. For antibiotics with complete utilization data in both the empirical and definitive therapy phases (n=7 pairs), the Shapiro-Wilk test was used to confirm normality of the paired differences, followed by a paired Student's t-test to assess the shift in ACI between empirical and definitive therapy. A p<0.05 with a 95% confidence interval (CI) was considered statistically significant.

## Results

Demographic profile

Of the 130 patients analyzed, 96 (73.8%) were male, and 34 (26.3%) were female. The mean age was 63.65±11.54 years. The majority of patients (n=46, 35.4%) were in the 61-70-year age group. Approximately 72 patients (55.4%) were above 65 years of age, meeting the age criterion for hospital admission in CAP. Table 1 presents the age-wise distribution.

**Table 1:** Age-wise distribution of community-acquired pneumonia patients (n=130).

Age (years)	Frequency (n)	Percentage
21-30	2	1.5
31-40	2	1.5
41-50	9	6.9
51-60	39	30
61-70	46	35.4
71-80	23	17.7
81-90	9	6.9
Total	130	100

Microbiological findings

A definitive causative organism was identified in the majority of patients, while no pathogen could be isolated in a minority of cases. *Streptococcus pneumoniae* was the predominant bacterial isolate, followed by Gram-negative organisms, particularly *Klebsiella pneumoniae* and *Escherichia coli.* *Staphylococcus aureus* and *Pseudomonas aeruginosa *were isolated less frequently. The detailed distribution of bacterial isolates is presented in Table 2.

**Table 2:** Distribution of microbial pathogens isolated in CAP patients (n=130). CAP: community-acquired pneumonia

Pathogen	Frequency (n)	Percentage
Streptococcus pneumoniae	40	30.8
Klebsiella pneumoniae	27	20.8
Escherichia coli	24	18.5
Staphylococcus aureus	8	6.2
Pseudomonas aeruginosa	2	1.5
No organism isolated	29	22.3
Total	130	100

Antibiotic susceptibility patterns

Linezolid demonstrated 100% sensitivity across all isolates where tested (n=40). Carbapenems (imipenem and meropenem) showed high sensitivity for *S. pneumoniae* (n=36, 90%), *K. pneumoniae* (n=26, 96%), and *E. coli* (n=19, 79%). Aminoglycosides (amikacin and gentamicin) showed moderate to good sensitivity. In contrast, ampicillin demonstrated the highest resistance rates (n=36, 90%) among *S. pneumoniae* isolates and (n=22, 81%) among *K. pneumoniae* isolates. Amoxicillin-clavulanic acid also showed substantial resistance. Table 3 presents detailed sensitivity and resistance data for all pathogens and antibiotic classes.

**Table 3:** Antibiotic susceptibility patterns of microbial pathogens isolated in CAP patients. S: sensitive; R: resistant; NA: not applicable; CAP: community-acquired pneumonia

Antibiotic	*S. pneumoniae* (n=40)		*K. pneumoniae* (n=27)		*E. coli* (n=24)		*S. aureus* (n=8)		*P. aeruginosa* (n=2)	
	S	R	S	R	S	R	S	R	S	R
Ampicillin	4	36	5	22	4	20	3	5	0	2
Amoxicillin-clavulanic acid	7	33	12	15	8	16	2	4	0	2
Piperacillin-tazobactam	15	25	10	17	14	10	3	5	2	0
Ceftriaxone	14	26	17	10	11	13	3	5	2	0
Ciprofloxacin	22	15	15	11	14	10	3	4	1	1
Levofloxacin	26	14	15	12	20	4	4	4	1	1
Doxycycline	23	17	16	11	15	9	4	4	2	0
Azithromycin	23	12	15	12	18	6	4	1	1	0
Amikacin	34	6	18	9	19	5	6	2	2	0
Gentamicin	35	5	15	12	16	8	4	1	2	0
Imipenem	36	4	26	1	19	5	NA	NA	2	0
Meropenem	36	4	26	1	19	5	NA	NA	0	0
Linezolid	40	0	27	0	19	5	7	0	2	0

Comorbid conditions

The most prevalent comorbidities were diabetes mellitus (n=37, 28.5%), chronic obstructive pulmonary disease (COPD) (n=32, 24.6%), and cardiovascular disease (n=25, 19.2%). A chi-square test of independence was conducted to evaluate the overall distribution of comorbidities (cardiovascular, COPD, diabetes mellitus, or none) between male and female patients.

Post-hoc analysis using adjusted standardized residuals revealed that this overall significance was driven by a stark difference in COPD prevalence; 31.2% of males (30/96) presented with COPD compared to only 5.9% of females (2/34). There were no statistically significant differences between genders in the rates of cardiovascular disease, diabetes mellitus, or those presenting with no comorbidities (Table 4).

**Table 4:** Distribution of comorbidities by gender in CAP patients. CAP: community-acquired pneumonia; COPD: chronic obstructive pulmonary disease

Gender	Cardiovascular, n (%)	COPD, n (%)	Diabetes mellitus, n (%)	None, n (%)
Male (n=96)	18 (18.8)	30 (31.2)	24 (25.0)	24 (25.0)
Female (n=34)	7 (20.6)	2 (5.9)	13 (38.2)	12 (35.3)
Total (n=130)	25 (19.2)	32 (24.6)	37 (28.5)	36 (27.7)

Empirical and definitive antibiotic therapy

The most commonly prescribed empirical antibiotic was amoxicillin-clavulanic acid (n=73, 56.2%), followed by levofloxacin (n=33, 25.4%), ceftriaxone (n=16, 12.3%), ofloxacin (n=6, 4.6%), and piperacillin-tazobactam (n=2, 1.5%). Following culture-sensitivity reports, definitive therapy was dominated by levofloxacin (n=33, 25.4%), with azithromycin (n=9, 6.9%), vancomycin (n=5, 3.8%), and meropenem (n=18, 13.8%) also frequently used. The most common combination in empirical therapy was amoxicillin-clavulanic acid with azithromycin (43% of combination prescriptions). Overall, poly-antibiotic therapy was prescribed in 115 patients (88.4%).

Antibiotic consumption index (ACI)

In empirical therapy, amoxicillin-clavulanic acid recorded the highest ACI (14.4 DDD/100 bed-days), followed by azithromycin (12.6 DDD/100 bed-days). In definitive therapy, levofloxacin exhibited the highest ACI (14.8 DDD/100 bed-days), followed by meropenem (13.0 DDD/100 bed-days). Total DDD consumption was significantly higher during definitive therapy (52.24 DDD/100 bed-days) compared to empirical therapy (35.34 DDD/100 bed-days).

A paired analysis was conducted to evaluate how the consumption of individual antibiotics shifted when transitioning from the empirical phase to the definitive phase. To maintain the paired structure of the data, the analysis was restricted to the antibiotics with complete tracking metrics across both phases (n=7 pairs).

A normal distribution of the differences was confirmed via the Shapiro-Wilk test (p=0.734). A paired t-test revealed that while there was an overall mean increase from the empirical phase (mean=5.05 DDD/100 bed-days) to the definitive phase (mean=6.73 DDD/100 bed-days), this total volume shift was not statistically significant (t=0.561, df=6, p=0.5948).

Crucially, the paired data demonstrates highly targeted clinical behavior - outpatients or mild-case oral frontline drugs like amoxicillin-clavulanic acid and azithromycin dropped sharply once definitive targets were established. Concurrently, heavy-hitter institutional restricted drugs like meropenem and levofloxacin saw massive spikes in paired volume, effectively balancing out total institutional consumption metrics. The remaining eight therapies listed in the dataset could not be included in the formal paired hypothesis testing due to a lack of baseline empirical utilization metrics. Table 5 provides ACI values for all agents across both phases.

**Table 5:** Antibiotic consumption index (ACI) for empirical and definitive therapy. ATC: Anatomical Therapeutic Chemical code; DDD: defined daily dose

Drug	ATC code	DDD (g)	ACI empirical (DDD/100 bed-days)	ACI definitive (DDD/100 bed-days)
Amoxicillin-clavulanic acid (oral)	J01CR50	1	14.4	5.52
Amoxicillin-clavulanic acid (parenteral)	J01CA12	3	-	-
Piperacillin-tazobactam	J01CA12	14	0.14	0.90
Ceftriaxone	J01DD04	2	0.94	6.17
Cefoperazone	J01DD12	4	-	0.10
Azithromycin (oral)	J01FA10	0.3	12.6	5.14
Clarithromycin (oral)	J01FA09	0.5	-	0.94
Ofloxacin	J01MA01	0.4	0.62	1.60
Levofloxacin	J01MA12	0.5	6.47	14.8
Moxifloxacin	J01MA14	0.4	-	0.15
Doxycycline	J01AA02	-	-	0.18
Amikacin	J01GB06	1	-	0.83
Meropenem	J01DH02	2	0.18	13
Vancomycin	J01XA01	2	-	2.40
Linezolid	J01XX08	1.2	-	0.15

Prescribing indicators

The mean number of drugs prescribed per patient was 7.67±2.17. The mean number of antibiotics per prescription was 2.66±1.89. Approximately 32% of drugs were prescribed using generic names. The mean duration of empirical therapy was 3.42±0.80 days, of definitive therapy 4.47±0.87 days, and the mean total hospital stay was 7.35±1.63 days. Intravenous administration was the predominant route (93.5% of antibiotic prescriptions in empirical therapy; 76.2% in definitive therapy).

## Discussion

This study presents a comprehensive analysis of both the microbiological profile and antimicrobial prescribing patterns in 130 hospitalized CAP patients from Western Uttar Pradesh, India. The study provides local data on pathogen distribution, antimicrobial susceptibility, and antibiotic utilization that may help inform future antimicrobial stewardship initiatives.

The mean patient age of 63.65±11.54 years and male predominance (73.8%) are consistent with published literature from India and globally. Like a study by Tilahun et al. [14], which reported older male preponderance in CAP cohorts from resource-limited settings, Chalmers also noted that age and male sex are independent predictors of CAP severity requiring ICU admission [15]. The predominance of older male patients observed in our study is consistent with previous reports from India and other countries. Although previous studies have suggested that factors such as smoking, COPD, and age-related decline in immune function may contribute to the higher burden of CAP among older males, these variables were not specifically evaluated in the present study.

*S. pneumoniae* was the most frequently isolated pathogen (30.8%), in keeping with national data reported by Menon et al. and international findings by Jain et al. [16,17]. Notably, a substantial proportion of isolates were Gram-negative organisms, *K. pneumoniae* (20.8%) and *E. coli* (18.5%), suggesting a shift in the local microbiological landscape. Although *S. pneumoniae* continues to be the leading cause of CAP worldwide, our findings demonstrate a relatively high proportion of Gram-negative pathogens. Like the study by Shah et al., which documented a similar Gram-negative burden in hospitalized Indian CAP patients, Goel et al. also reported this trend in an intensive care cohort [5,6]. Mehrad et al. emphasized the clinical significance of this finding, noting that Gram-negative pathogens carry broader antibiotic resistance profiles and require empirical regimens distinct from those used for typical CAP pathogens in high-income settings [18].

The susceptibility pattern observed in this study has important implications for empirical antibiotic selection. Linezolid demonstrated 100% sensitivity, making it a reliable option for severe CAP with resistant Gram-positive organisms. However, as Leekha et al. emphasized, reserve agents such as linezolid should be preserved given their cost and the risk of accelerating resistance if used empirically [19]. Carbapenems and amikacin remain effective against Gram-negative organisms, while resistance to ampicillin and amoxicillin-clavulanic acid was strikingly high. Like the study by Menon et al., which reported high beta-lactam resistance in Indian CAP isolates, Jenkins et al. also found that high rates of first-line antibiotic resistance necessitated escalation to broad-spectrum agents, rendering these first-line agents unsuitable as empirical monotherapy in similar resource-limited settings [16,20]. High resistance to ampicillin and amoxicillin-clavulanic acid suggests that these agents may no longer provide adequate empirical coverage for all hospitalized CAP patients in our setting.

Regarding antibiotic utilization, amoxicillin-clavulanic acid was the dominant empirical agent (n=73, 56.2%). Like the study by Kotwani et al. [21], which reported amoxicillin-clavulanic acid as the most frequently prescribed empirical antibiotic in hospitalized CAP patients in New Delhi, Nayar et al. also noted its widespread use in Indian tertiary care settings [22]. The high ACI for amoxicillin-clavulanic acid (14.4 DDD/100 bed-days), followed by significant escalation to levofloxacin (ACI: 14.8) and meropenem (ACI: 13.0) during definitive therapy, illustrates a predictable failure cascade driven by high first-line resistance. The significant increase in total DDD from empirical to definitive therapy has been replicated by Ravi et al. in a Northern Indian cohort [23]. This pattern suggests that culture-guided optimization of therapy was practiced in a substantial proportion of patients and highlights the importance of timely microbiological diagnosis in antimicrobial stewardship.

The high rate of poly-antibiotic prescriptions (88.4%) and mean antibiotic load of 2.66 per prescription raise concerns about irrational prescribing. Martin-Loeches et al. endorsed combination therapy in their European Respiratory Society (ERS)/European Society of Intensive Care Medicine (ESICM)/European Society of Clinical Microbiology and Infectious Diseases (ESCMID)/Latin American Thoracic Association (ALAT) guidelines only for severe CAP requiring ICU admission, and routine use in moderate-severity inpatients adds drug burden without proven clinical benefit [24]. The low rate of generic prescribing (32%) and the predominance of intravenous therapy, even when oral bioequivalents are available, reflect prescribing habits amenable to stewardship interventions. Shrayteh et al. demonstrated that intravenous-to-oral switch protocols are safe, cost-effective, and widely feasible; Cyriac and James also found that timely oral switching reduced hospital stay without compromising outcomes in comparable settings [25,26].

International comparison reveals that guideline adherence in Norway, Malaysia, and the Netherlands ranges from 56-73%, with a general preference for narrow-spectrum agents (Table 6). In contrast, our setting demonstrates heavy reliance on broad-spectrum beta-lactam combinations empirically, driven by a combination of high Gram-negative burden, local resistance patterns, and entrenched prescribing culture. Like the study by Waagsbø et al., which documented that structured antibiotic stewardship efforts during the COVID-19 pandemic significantly shifted prescribing toward narrower-spectrum agents in Norwegian hospitals, similar guideline-concordant prescribing programs represent a major opportunity for tertiary care centers in India [27].

**Table 6:** International comparison of antimicrobial prescribing patterns in CAP. CAP: community-acquired pneumonia

Country	Antimicrobial prescribing	Microbiological confirmation	Guideline adherence	Reference
Norway (2016, 2021)	Narrow-spectrum beta-lactam 54%; fluoroquinolones 12%; broad-spectrum cephalosporins 34%	*S. pneumoniae* 23.8%; *H. influenzae* 22.4%; *M. catarrhalis* 11.9%	56.2%	Waagsbø et al. (2022) [27]
Malaysia (2022)	Narrow-spectrum beta-lactam 52.6%	Not specified	60.8%	Loong et al. (2022) [28]
Netherlands (2015)	Beta-lactam 57%; beta-lactam+macrolide 20.8%; fluoroquinolone 22.3%	Not available	72.8%	Postma et al. (2015) [29]

The presence of diabetes mellitus (28.5%) and COPD (24.6%) as the dominant comorbidities is consistent with findings from Maharashtra and other Indian settings. Like the study by Ginsburg and Klugman, which observed that immunocompromising comorbidities significantly altered pathogen profiles and antibiotic requirements, both conditions are known to predispose patients to Gram-negative and drug-resistant infections, further supporting the clinical rationale for culture-guided definitive therapy in all hospitalized CAP patients with significant comorbidities [8].

A key limitation of this study is the inability to detect viral and atypical pathogens (Mycoplasma, Chlamydophila, Legionella), which may account for a proportion of culture-negative cases (22.3%). The study was conducted prior to the COVID-19 pandemic wave, which limits direct applicability to pandemic-era practice. Additionally, the short study duration precluded assessment of seasonal variation.

## Conclusions

This integrated study of bacteriological profile, antibiotic susceptibility, and drug utilization in CAP provides a multidimensional foundation for evidence-based prescribing in Western Uttar Pradesh. *Streptococcus pneumoniae* was the most frequently isolated pathogen, although a substantial proportion of infections were caused by Gram-negative organisms. The observed antimicrobial susceptibility patterns and antibiotic utilization trends highlight the importance of local microbiological surveillance in guiding empirical therapy and optimizing subsequent culture-directed treatment. These findings provide baseline institutional data that may support future antimicrobial stewardship initiatives and periodic updates of local antibiograms. Further multicenter studies with larger sample sizes are warranted to validate these findings and improve the generalizability of the results.
